# The Two-Stage Ensemble Learning Model Based on Aggregated Facial Features in Screening for Fetal Genetic Diseases

**DOI:** 10.3390/ijerph20032377

**Published:** 2023-01-29

**Authors:** Jiajie Tang, Jin Han, Bingbing Xie, Jiaxin Xue, Hang Zhou, Yuxuan Jiang, Lianting Hu, Caiyuan Chen, Kanghui Zhang, Fanfan Zhu, Long Lu

**Affiliations:** 1 School of Information Management, Wuhan University, Wuhan 430072, China; 2 Institute of Pediatrics, Prenatal Diagnostic Center, Guangzhou Women and Children’s Medical Center, Guangzhou Medical University, Guangzhou 510180, China; 3 Graduate School, Guangzhou Medical University, Guangzhou 511436, China; 4 Medical Big Data Center, Guangdong Provincial People’s Hospital, Guangzhou 510080, China; 5 Guangdong Cardiovascular Institute, Guangdong Provincial People’s Hospital, Guangzhou 510080, China; 6 Center for Healthcare Big Data Research, The Big Data Institute, Wuhan University, Wuhan 430072, China; 7 School of Public Health, Wuhan University, Wuhan 430072, China

**Keywords:** fetal genetic disease, obstetrics and gynecology ultrasound, fetal facial, deep learning, ensemble learning

## Abstract

With the advancement of medicine, more and more researchers have turned their attention to the study of fetal genetic diseases in recent years. However, it is still a challenge to detect genetic diseases in the fetus, especially in an area lacking access to healthcare. The existing research primarily focuses on using teenagers’ or adults’ face information to screen for genetic diseases, but there are no relevant directions on disease detection using fetal facial information. To fill the vacancy, we designed a two-stage ensemble learning model based on sonography, Fgds-EL, to identify genetic diseases with 932 images. Concretely speaking, we use aggregated information of facial regions to detect anomalies, such as the jaw, frontal bone, and nasal bone areas. Our experiments show that our model yields a sensitivity of 0.92 and a specificity of 0.97 in the test set, on par with the senior sonographer, and outperforming other popular deep learning algorithms. Moreover, our model has the potential to be an effective noninvasive screening tool for the early screening of genetic diseases in the fetus.

## 1. Introduction

According to a study conducted in 1988, a disease with a significant genetic component is likely to affect at least 7.9 percent of newborns [1]. There are approximately 7000 known genetic diseases, with 30% to 40% of cases causing facial and skull abnormalities, particularly in Down syndrome, the most common genetic disease [2,3]. Genetic diseases are clinically linked to severe cardiovascular, immune, endocrine, and neurodevelopmental risks, affecting the quality of life of patients and their families [4]. Medical institutions have recently used Down syndrome screening, maternal plasma fetal cell-free fetal DNA (cffDNA), and expanded non-invasive prenatal testing (NIPT) to screen for genetic disease [5]. However, the ability of these screening methods to detect genetic diseases is limited: there is no effective screening for fetal monogenetic syndrome; these methods are time-consuming and laborious; and they may be unworkable in many countries and regions with underdeveloped healthcare models.

With the continuous advancement of ultrasonic diagnosis techniques and the accumulation of clinical experience, prenatal ultrasound has become widely used in prenatal genetic disease screening owing to the diverse advantages it offers, including real-time acquisition, low costs, no radiation, and noninvasiveness [6]. There are a large number of popular sonographic features, such as nuchal translucency (NT), nasal bone (NB), cleft lip, palatine jaw, and frontal bone, that can be used to screen for genetic diseases. In 2010, Ettema and his team found that in 302 cases of trisomy 21, 171 cases of trisomy 18, and 69 cases of trisomy 13, 14.2% of the trisomy 21 fetuses had abnormal skull morphologies, of which 41.5% of the trisomy 18 fetuses had abnormal facial contours, and 65.2% of the trisomy 13 fetuses had a cleft lip and palate [7]. Some studies reveal that 65% of trisomy 21 fetuses have an absent or short nasal bone according to the sonographic examination between 15 and 24 weeks of gestation [8,9,10,11,12]. With the development of craniofacial genetics, many rare genetic diseases have also been shown to have specific facial features, such as the broad nasal bridge in 15q11-q13 duplication syndrome, the maxillary hypoplasia in 17q22 microdeletion, the wide and depressed nasal bridge in Helsmoortel-van der AA syndrome, etc. [13,14,15]. These studies show the specificity of fetal facial features. However, the recognition of nonclassical phenotypes or ultra-rare syndromes is limited by the experience of the individual expert, making it critical to use new methods to help analyze fetal ultrasound indicators.

Computer vision has long been concerned with medical image analysis issues [16]. Deep learning has provided a new impetus to computer vision research [17,18,19]. In fetal ultrasound, deep learning has been used for standard plane recognition, standard plane localization, and screening for structural abnormalities [20,21,22,23,24,25]. If a computer vision system can be developed to recognize the facial information of normal and abnormal fetuses, it will be useful to help screen high-risk fetuses. Given the prevalence of prenatal ultrasound examinations in medical institutions of all levels around the world, well-developed deep learning models based on sonographic fetal facial images have the potential to alleviate the shortage of expertise in primary care and improve genetic disease screening accuracy.

In this study, we developed Fgds-EL (Fetal genetic disease-Ensemble Learning), a two-stage ensemble learning model that used a convolutional neural network (CNN) and Random Forest (RF) to automatically screen for genetic diseases in fetal ultrasound imaging. The Fgds-EL uses an aggregate of fetal facial regions to improve performance and robustness. Additionally, we analyzed the screening performance of subnetworks from Fgds-EL and show the contribution of image regions to the decision by interpretable techniques [26]. Moreover, we compare the performance of the Fgds-EL to that of sonographers of different levels.

The main contributions of this paper are as follows: (1) the development of a two-stage ensemble learning model based on fetal facial, named Fgds-EL, for robust and accurate screening genetic diseases; (2) evaluating the performance of Fgds-EL in screening for fetal genetic diseases from ultrasound images; (3) the decision-making area of the Fgds-EL is visualized using the heat map to discover the anatomical structures related to genetic diseases; and (4) comparing the performance of the Fgds-EL to that of sonographers.

## 2. Materials and Methods

### 2.1. Data Acquisition

In this study, we collected ultrasound fetal profile images from around 1000 pregnant women with an age range of 23–38 years who underwent prenatal diagnosis at the Guangzhou Women and Children’s Medical Center. We excluded (*n* = 333) from the initial list of 1000 cases, those without genetic results, 3D volume images and complete clinical data. Thus, our dataset contained 556 normal pregnancies and 111 pregnancies with genetic anomalies. All examinations have been performed and diagnosed before the research by one or two ultrasound specialists with more than five years of experience. Overall, our datasets contain 845 normal pregnancy images and 275 genetic disease images from 667 cases, with a gestational age distribution of 14.8 ± 2.6 (range 11–27). Details on the development set and test set are described in Table 1.

Finally, we used a program script to randomly divide positive cases into training and test sets at a ratio of 3:1. The training set includes 189 images of 74 positive cases, and the test set includes 63 images of 37 positive cases. In order to balance the proportion of samples in the test set, we randomly divided the negative cases into the training set and the test set at a ratio of 9:1. Therefore, the training set includes 587 images of 56 negative cases, and the test set includes 93 images of 500 negative cases. It is worth noting that this dataset is divided at the case level rather than the image level to observe a more realistic screening performance. 

This study was approved by the Institutional Review Board of the Guangzhou Women and Children’s Medical Center (473B01, 2021). Each participant signed an informed consent form.

### 2.2. Date Preprocessing

At first, we converted all the images into grayscale images. Following that, we rescaled the images to 400 × 440 pixels in size and normalized the pixel values to a range of 0 to 1. In addition, we augmented the training data set with distortion, zoom in, tilt, zoom out, and crop. All preprocessing steps make use of open-source Python libraries: OpenCV, scikit-image, and NumPy.

Following data preprocessing, a total of 20,000 images, including 10,000 normal pregnancy images and 10,000 genetic disease images, are used to develop Fgds-EL.

### 2.3. Ensemble Learning Architecture

**Figure 1:**
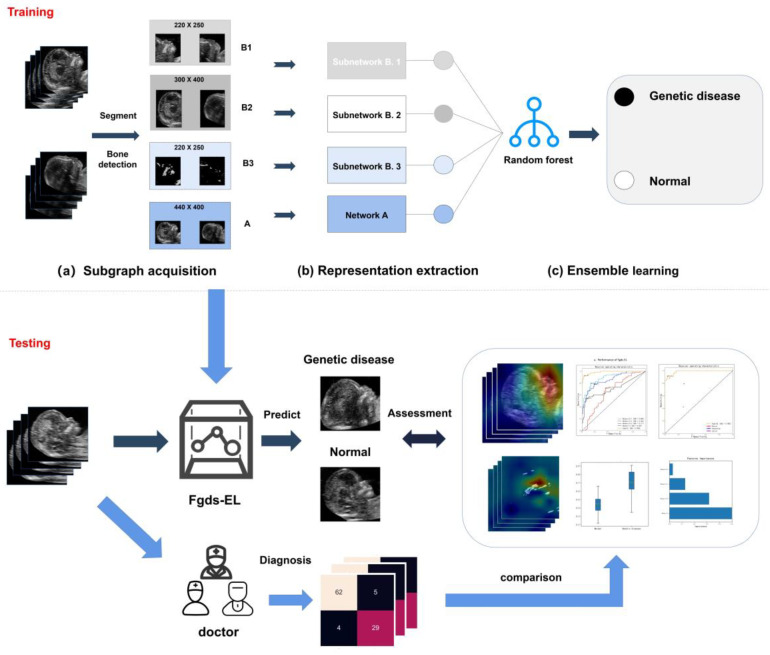
Workflow of the deep learning model for screening fetal genetic diseases. First, the fetal facial image is extracted into four subgraphs by automatic segmentation and bone detection and input into four networks, respectively. Then, the joint prediction probability is obtained using RF on the prediction results of the four networks, and finally, the risk score and heatmap corresponding to this image is obtained.

Both local and global facial features have been considered as significant variations for the detection of genetic diseases. Figure 1 gives a block diagram representation of the proposed model. Our deep learning model is named Fgds-EL (Fetal Genetic Disease Screening Model based on Ensemble Learning). We design a deep learning architecture structured into three parts, which perform global facial feature extraction (Network A), local feature extraction (Network B), and genetic disease risk estimation (RF).

Our deep learning architecture is inspired by an experiment conducted to predict the genetic diseases of the whole face, including the upper face, middle face, and lower face [27,28]. In our proposed model, subgraphs B1 and B2 were initially cropped from the facial images with the help of an automated program. Subgraph B3 was cropped from the images with the help of a bone detection algorithm that uses threshold segmentation [29]. These cropped pictures are divided into three parts, which will be fed into different networks along with the full face information for extracting the essential representation of the facial features. The extracted representations are then aggregated into a single representation vector. After that, an ensemble learning model is trained on these representations for further classification. The entire framework can be divided into three parts: subgraph acquisition (segmentation), fetal facial representation extraction, and ensemble learning.

#### 2.3.1. Subgraph Acquisition

In the subgraph acquisition part (Figure 1a), the ultrasound image is extracted as four subgraphs. Subgraph A contains the whole face, including the upper face, middle face, and lower face. Subgraph B1 contains the middle and lower face. It includes 220 × 250 pixels from the facial image. Subgraph B2 contains the upper face and hindbrain. It includes 300 × 400 pixels from the facial image. Subgraph B3 is the bone detection subgraph, in which we use the detection method proposed by Lai et al. to extract the features of the nasal bone and jaw [29]. An example of the various subgraphs extracted from a fetal profile is shown in subgraph acquisition in Figure 1.

#### 2.3.2. Fetal Facial Representation Extraction

In subgraph A, Network A identified facial differences to estimate the probability of a patient presenting with a genetic disease and the probability of the patient not presenting with a genetic disease from the facial image of size 440 × 400 pixels. Network B embedded three networks (B.1, B.2, and B.3) with the architectures described in Figure 1b. Network B.1, B.2, and B.3 are connected to subgraphs B1, B2, and B3. They identified facial differences to estimate the probability of a patient presenting with a genetic disease and the probability of the patient not presenting with a genetic disease. ReLU (Rectified Linear Unit) activation functions were used after each filter operation. A ReLU function was used in the final layer to ensure that the probabilities of the two classes were disjoint. All pooling operations used two strides to decrease the data size to half on each spatial dimension. Each network is trained separately, the outputs of each network’s last neural network layer are selected as the probability of genetic disease, and we simply concatenate them together to form multiple dimensional features. We used ResNet-50 as Network B.1 and ResNet-18 as Network A. Considering that the key information in Subgraph B2 and Subgraph B3 are low-level image features, we constructed Networks B.2 and B.3 using the residual module. The structures of Network B.2 and Network B.3 are shown in Figure S1.

#### 2.3.3. Ensemble Learning

We use RF as the stacking model of sub-networks in ensemble learning, and RF fuses the disease probability of each network to estimate the comprehensive probability of a patient presenting with a genetic disease and the comprehensive probability of a patient not presenting with a genetic disease. To prevent overfitting, we adjusted the parameters of the training set. The number of parameters of the decision tree is 43, the maximum depth of the decision tree is 3, and the other parameters are the default parameters.

Networks A, B, and RF were trained in two stages. First, Networks A and B were used to predict the disease probability of each network. Following that, RF is used to combine each network’s prediction probability to obtain the overall classification performance. In the CNN model, at least one residual block is added to each network, and batch normalization optimization is carried out in each convolution layer. Learning rate optimization was performed using the Adam optimizer, with an initial learning rate of 1 × 10^−5^. During the training process, the callback function is used to keep an eye on the validation set loss function, and the best model is saved.

We implemented and trained Fgds-EL, and all the functionality, experiments, and analysis were implemented using Python (NumPy 1.16, for array manipulation; opencv 4.1, scikit-image 0.17, imageio 2.8, and Pillow 7.1 for image operations; and scikit-learn 0.19 for performance quantification) and Google Tensorflow 2.0 (for the implementation of the deep learning architecture).

### 2.4. Performance Evaluation

To comprehensively evaluate the excellent performance of our proposed Fgds-EL model, we trained the other models, including classic convolution network models, such as ResNet [30], VGG [31], and DenseNet [32,33]. At the same time, in order to further analyze the performance of our method, we also compare it with other recent deep learning models, such as ResNest50 [34], EfficientFormer [35], Swin Transformer [36], and RepLKNet [37]. Since the proposed model combines several CNN models, we also measure the processing speed of a single sample and compare it with other models in the results section. The CPU of our experimental platform is an Intel Core i7-11800H, and the GPU is a NVIDIA GeForce RTX 3080 Laptop.

In the statistical analysis, six quantitative indicators are used to evaluate the screening performance, such as accuracy (ACC), sensitivity (SEN), specificity (SPEC), and F1 score. F1 score, also known as balanced F score, is defined as the harmonic average of the precision and recall rates. Additionally, we use the receiver operating characteristic (ROC) curve and the area under the receiver ROC curve (AUC) to assess the screening performance between different models and networks. Additionally, ROC analysis is performed to determine the optimal operating thresholds by using the outputs of the models on the tuning dataset. We also use the distribution of risk scores related to the genetic disease determined by Fgds-EL in test sets.

### 2.5. Performance Comparison between the Fgds-EL and Prenatal Diagnostic Doctor

To compare the Fgds-EL’s performance to that of a doctor, we recruited three sonographers with varying levels of clinical experience (a junior sonographer with three years of experience, a junior professional sonographer with seven years of experience, and a senior with 15 years of experience). Fgds-EL and each sonographer were given 100 images from the test set and were asked to independently determine whether the fetus had certain genetic diseases. 

### 2.6. Heat Map Generation

Grad-CAM is used to visualize the decision-making ability of our model by adding a visualization layer to the CNN model [26]. This method uses target concept gradients to generate a localization map highlighting key image regions for concept prediction. Redder regions indicate more important predictions. A heat map is generated using this tool to interpret the Fgds-EL’s rationale for distinguishing between normal fetal facial development and genetic diseases of the face. 

Instead of retraining the model or making changes to it, Grad-CAM’s approach simply takes a single image into the network to extract the top layer feature maps and uses backpropagation to compute the gradients to generate a category gradient matrix. In this way, we can obtain a feature map associated with the category information, and then overlay it with the original image to obtain a heat map that can explain the model’s decision logic.

## 3. Results

### 3.1. Dataset Characteristics

A total of 932 images (including 680 normal pregnancy images and 252 genetic disease images) are retrieved for developing the ensemble learning model. Following data preprocessing, a total of 20,000 images, including 10,000 normal pregnancy images and 10,000 genetic disease images, are used to develop Fgds-EL, and 158 images (including 94 images of normal pregnancies and 63 images of genetic diseases) are used to test our model. In the original dataset, the top three genetic diseases in our datasets are Down syndrome (55/252, 21.8%), trisomy 18 (80/252, 30.9%), and trisomy 13 (42/252, 16.4%). The other genetic diseases (e.g., monogenic genetic disease and Turner syndrome) are 30.9% (75/252), including Turner syndrome, 1q21.1 microdeletion, 15q11-q13 duplication syndrome, 15q26.1-q26.3 deletion and 20p13 duplication, Helsmoortel-van der AA syndrome, 4P syndrome, etc. Details on the development set and test set are described in Table 1.

### 3.2. Performance of the Fgds-EL and Each Subnetwork

**Figure 2:**
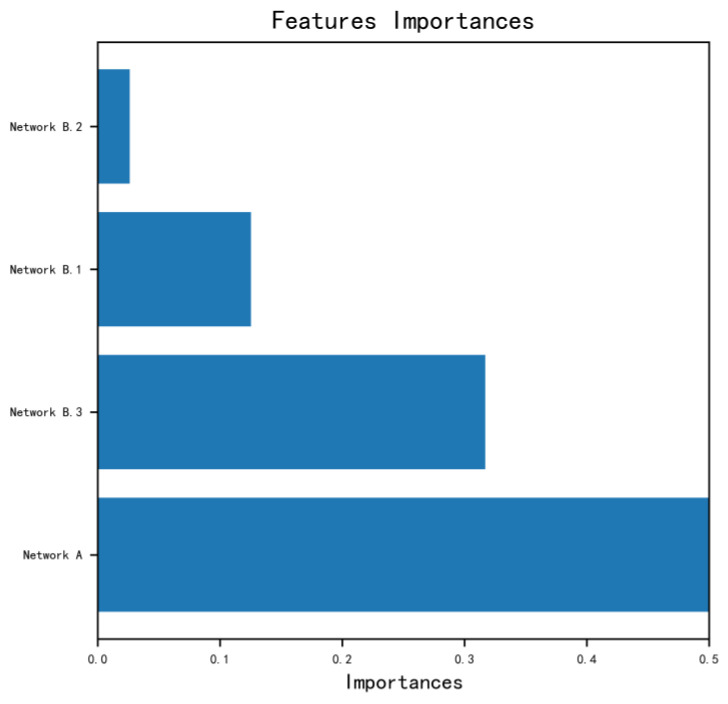
The feature importance obtained from ensemble learning.

**Figure 3:**
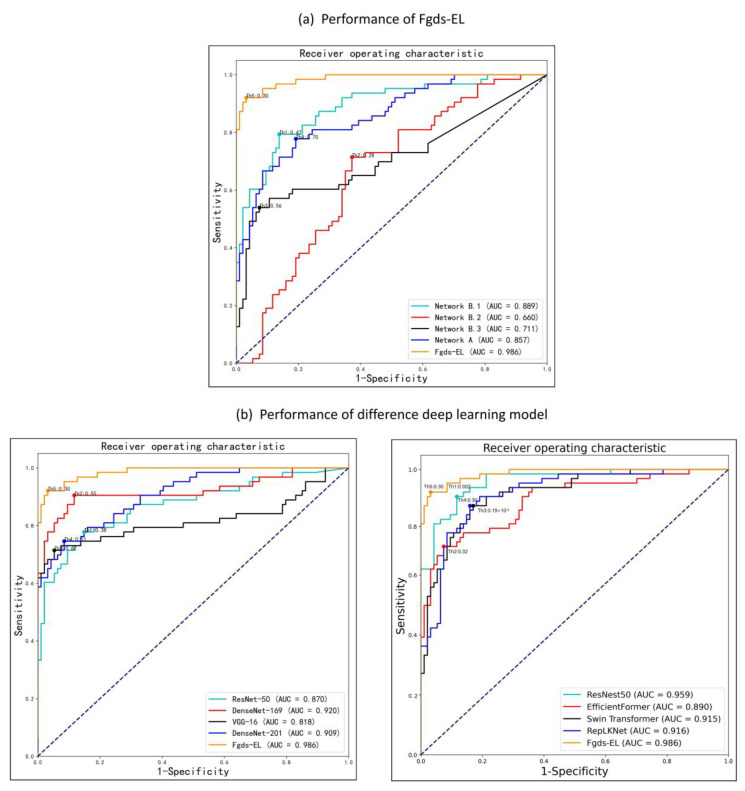
Performance of different deep learning algorithms in discerning fetal genetic diseases. (**a**) The receiver operating characteristic (ROC) curves of the different sub-networks and Fgds-EL in the test set. (**b**) The ROC curves of the different deep learning algorithms in the test set. AUC is the area under the ROC curve.

The development and validation processes of the Fgds-EL are shown in Figure 1. It uses an aggregation of fetal facial regions to improve performance and robustness. The fetal facial images are extracted into four subgraphs by an automated program. To examine how each subgraph contributes to the ensemble learning model and genetic disease screening, we evaluate each subgraph’s performance separately in comparison to the ensemble learning model. The feature importance obtained from ensemble learning is shown in Figure 2. It is used to show the contribution of each subgraph to Fgds-EL. We draw the ROC curve obtained from the subnetworks (Networks A, Networks B.1, Networks B.2, and Networks B.3) in Fgds-EL training. For comparison, the ROC curve from ensemble learning is also shown in Figure 3a.

For screening genetic diseases, the optimal algorithm Fgds-EL achieves an area under the receiver operating characteristic curve (AUC) of 0.986, a sensitivity of 0.92, and a specificity of 0.97 in the test set. We also calculate the performance of each subnetwork to find out how different parts of the fetal face affect the screening for genetic diseases. Network A achieves the best performance in the subnetwork, including an AUC of 0.857, a sensitivity of 0.76, and a specificity of 0.81 by training subgraph A. Network B.1 achieves an AUC of 0.889, a sensitivity of 0.78, and a specificity of 0.86 by training subgraph B1, and Network B.2 obtains an AUC of 0.66, a sensitivity of 0.70, and a specificity of 0.63 by training subgraph B2. In the bone detection subgraph, Network B.3 attains an AUC of 0.711, a sensitivity of 0.52, and a specificity of 0.93 by training subgraph B3. Network B.1 shows the best performance in subnetworks, which indicates that subgraph B1 contains information that is essential for screening genetic diseases. Further information encompassing the F1 score of Fgds-EL and the subnetwork is also displayed in Table 2.

**Figure 4:**
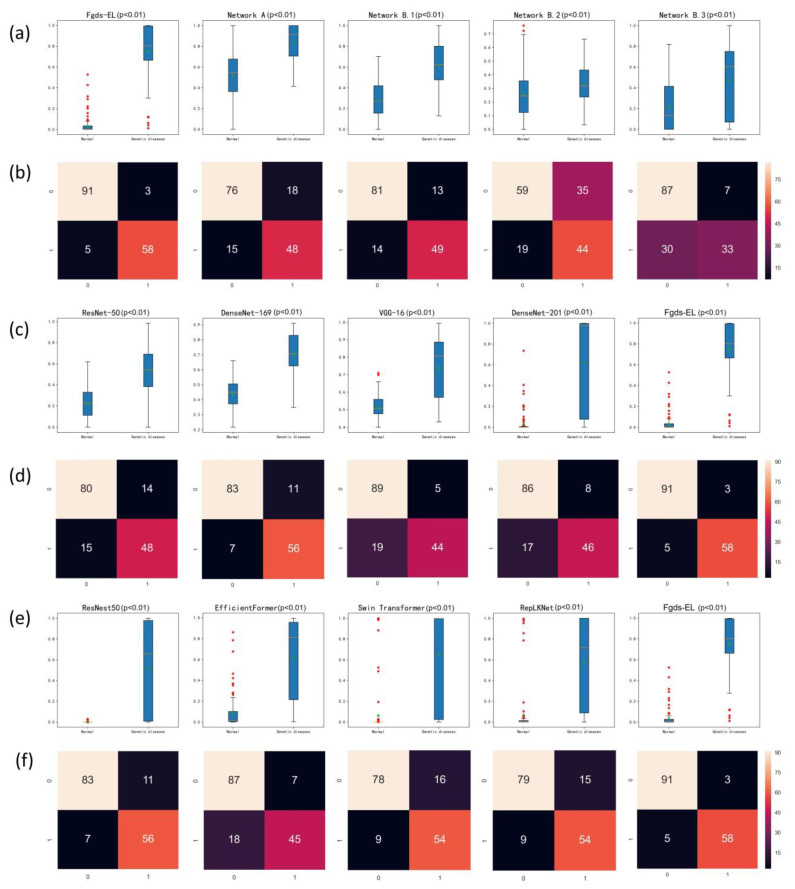
Risk scores (range 0–1) and confusion matrix predicted by the deep learning model for discerning fetal genetic diseases. Scores closer to 1 denote a higher probability of genetic diseases. The upper and lower bounds of the box refer to the 25th and 75th percentiles, and the line intersection in the box refers to the median. Green triangles and red stars represent outliers and mean values respectively. Whiskers refer to the full range of risk scores. In the confusion matrix, the horizontal coordinate is the predicted label, and the vertical coordinate is the true label. (**a**) The risk scores predicted by the different sub-networks and Fgds-EL in the test set. (**b**) The confusion matrix of the different sub-networks and Fgds-EL in the test set. (**c**,**e**) The risk scores predicted by the different deep learning models in the test set. (**d**,**f**) The confusion matrix of the different sub-networks and Fgds-EL in the test set.

The distribution of the predicted scores and the confusion matrices related to the categories determined by the Fgds-EL and sub-networks in test sets are shown in Figure 4a,b. With the threshold set at 0.3, the percentage of correctly classified images in genetic disease was 92.1% (58/63) in Fgds-EL. By comparison, those correctly classified were 76.2% (48/63) in Network A (threshold = 0.7), 77.8% (49/63) in Network B.1 (threshold = 0.47), 71.4% (45/63) in Network B.2 (threshold = 0.6), and 52.4% (33/63) in Network B.3 (threshold = 0.56), respectively.

### 3.3. Performance of the Fgds-EL and Other Deep Learning Algorithms

The screening results of the Fgds-EL for genetic diseases are shown in Table 1. Eight classic deep learning algorithms, DenseNet-169, DenseNet-121, ResNet-50, VGG-16, ResNest50, EfficientFormer, Swin Transformer, and RepLKNet, are used to train models to compare with Fgds-EL. The ROC curves of these algorithms in test sets are shown in Figure 3b, and the corresponding confusion matrices and the distribution of predicted scores are presented in Figure 4c–f, which indicates that our Fgds-EL is the optimal algorithm. The F1 score also shows that the features of different facial genetic diseases learned by the Fgds-EL are more identifiable than others. Further information encompassing AUC, sensitivities, specificities, and F1 scores of these algorithms and Fgds-EL is displayed in Table 2.

Since the proposed model combines several CNN models, we also measure the processing speed of a single sample. The results show that the model loading time of Fgds-EL is 2.638 s, and the model prediction time is 0.942 s, which is slightly higher than ResNet-50 and lower than DenseNet. In the recent deep learning model, Swin Transfomer has the fastest processing speed, while ResNest50 and RepLKNet have the slowest processing speeds. The processing speed of all the models is shown in Table 3.

### 3.4. Importance Score from Fgds-EL

RF is an ensemble learning classifier based on the decision tree with good model interpretability. Fgds-EL used an ensemble learning model based on a RF, which is stacked by networks A, B.1, B.2, and B.3. The importance score can reflect the contribution of each subgraph to Fgds-EL screening performance and help us understand the logic of the model. The importance scores of network A, network B.1, network B.2, and network B.3 are 0.53, 0.16, 0.03, and 0.32, respectively, as shown in Figure 2. This indicates that the bone detection subgraph (subgraph B3) promotes the performance of the Fgds-EL. At the same time, subgraph B1 enlarges and focuses on some facial features that are not visible in the original image. 

### 3.5. Fgds-EL Detects Facial Abnormalities by Subnetworks’ Heatmaps

**Figure 5:**
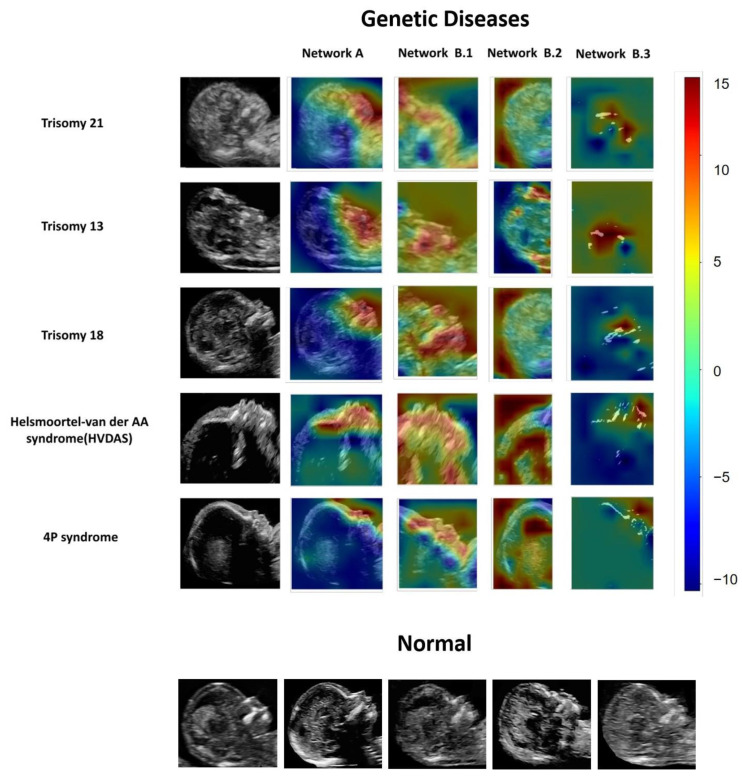
Example of fetal genetic diseases and corresponding heatmaps from Network A and Network B (B.1, B.2, B.3). The heatmap illustrates the importance of local areas within the image for classification. The importance value is scaled between −10 and 15, where a higher number indicates that the area is of higher importance for classifying the image as consistent with genetic diseases.

To explore the interpretability of the Fgds-EL in screening genetic diseases, we use the heatmaps created by Grad-CAM algorithms to visualize the regions that contributed most to the model’s decisions. We find that heatmaps highlight the mouth, nose, jaw, and forehead regions. Furthermore, the heatmap from the sub-network highlights some abnormalities of the facial features of genetic diseases in more detail. According to the heatmap, there are three findings shown as follows. (1) Network B3 is able to accurately identify information such as the nasal and jaw bones of the fetus, which are often overlooked in the analysis of the global image. (2) Network B2 focuses on the morphological features of the skull, and the flattened forehead of trisomy 13 is accurately identified by the heat map. (3) The heat map of Network B1 focuses on the richer information of facial details. Examples of images and corresponding heatmaps of genetic disease are shown in Figure 5.

As shown in Figure 5, the heatmap indicated that trisomy 21 exhibited the phenotypic characteristics of absent and hypoplastic nasal bones in the fetuses; trisomy 13 presents with holoprosencephaly, cyclopia, ethmocephaly, cebocephaly, or premaxillary agenesis; trisomy 18 is associated with facial features, such as cleft lip and jaw deformity; in the screening test of a Helsmoortel-van der AA syndrome, Fgds-EL accurately detected the prominent forehead, wide and depressed nasal bridge, and upturned nasal tip; and 4P syndrome has special facial features such as bulging eyebrows and eyes and a small jaw deformity. Other clinical findings are shown in the Supplementary Material.

### 3.6. Fgds-EL’s Performance Is on Par with the Senior Sonographers

**Figure 6:**
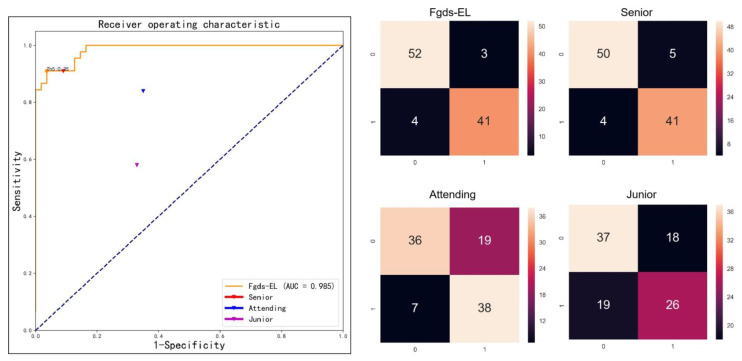
The ROC curves of the Fgds-EL and confusion matrix for screening for genetic diseases on the test set with three levels of sonographer performance (junior, attending, and senior) to compare. In the confusion matrix, the horizontal coordinate is the predicted label, and the vertical coordinate is the true label.

For screening genetic disease from the test set, the junior sonographer achieves an accuracy of 0.63 with a sensitivity of 0.42 and a specificity of 0.73, the attending achieves an accuracy of 0.74 with a sensitivity of 0.79 and a specificity of 0.72, and the senior obtains an accuracy of 0.91 with a sensitivity of 0.88 and a specificity of 0.93, while the Fgds-EL acquires an accuracy of 0.93 with a sensitivity of 0.91 and a specificity of 0.95. The performances of these three sonographers are presented in Table 4 and Figure 6.

## 4. Discussion

The goal of this study is to develop a significant ensemble learning algorithm (named Fgds-EL) to screen for genetic diseases in the fetus. The workflow of our deep learning model for screening fetal genetic diseases is shown in Figure 1. Figure 3 and Figure 4 show that Fgds-EL algorithms are effective at screening genetic diseases, outperforming ResNet, ResNest50, DenseNet, Swin Transformer, and other algorithms, and have an acceptable processing speed. In a comparative study of subnetworks, we discover that the Network B.1 developed by subgraph B.1 achieves the best performance (AUC 0.89, sensitivity 0.78, specificity 0.86). In light of our interpretable experiments, the features of the mouth and nose regions in subgraph B.1 have contributed a great deal to genetic disease facial manifestations. In our further study of ensemble learning, we show the importance of scores of sub-networks in Figure 2. The scores of network A, network B.1, network B.2, and network B.3 are 0.53, 0.13, 0.03, and 0.32, respectively. It illustrates that the nasal bone, jaw, and other knowledge learned by Network B.3 effectively enhance the performance of the Fgds-EL. In general, the results show that Fgds-EL has the potential to be an effective noninvasive screening tool for early screening of genetic diseases in the fetus, such as Down syndrome, reducing the use of medical resources (amniocentesis and NIPT) and improving the detection rate.

Chromosomal diseases are the most common genetic diseases and the leading causes of congenital disability. Abnormal facial manifestations usually accompany patients with chromosomal genetic syndromes [38,39,40,41]. With the rapid development of CNN technology, automatic facial recognition technology has been widely studied and developed in recent years. Facial recognition has become one of the most popular research topics in pattern recognition and image processing in the past three decades. The latest research has shown that facial analysis technology can improve clinicians’ ability to diagnose genetic syndromes. FDNA has developed a facial disease recognition system, termed “face2Gene” [27], which combines computational facial recognition with a clinical knowledge database. It uses deep learning algorithms to aid pediatricians in the diagnosis of genetic syndromes through image recognition. This has attracted extensive attention in clinical genetics. Porras et al. developed a deep learning system including facial morphology detection, using facial images to evaluate a child’s risk of presenting with a genetic syndrome [28]. These studies use children’s facial photos to diagnose genetic syndromes. They have a high clinical application, making it possible to construct an AI screening system for fetal face recognition.

Our current method calculates the risk of genetic diseases (including common aneuploidies, subchromosomal abnormalities, and monogenic diseases) by using ultrasonographic images of the head through a routine structural scan in the first and mid-trimester. This means that original images are readily available and do not increase the additional workload of ultrasound technicians. It has the potential to be widely applicable in remote or underdeveloped areas. We hope that the model will reduce the time it takes to refer patients with suspected genetic diseases and speed up the process of diagnosing them.

Deep learning is regarded as a high-risk decision due to the lack of interpretability in diagnosis, and is even referred to as “a black box.” To investigate this issue, we use the RF algorithm for ensemble learning and the importance score to visualize the contribution of subnetworks to the final decision. We also create heatmaps using the Grad-CAM technique [26] to visualize the regions that contribute the most to the classification of the model. The heatmap highlights high-contributing areas in images, such as the nose, mouth, forehead, and others. Notably, we also show the heatmap of each subgraph in the model, which is useful for understanding the basis of model evaluation from different perspectives and exploring abnormal facial features. As shown in Figure 1 and Figure 5, network A learns the entire image and has good recognition ability for global image features, but misses local features such as bone features. Network B.1 is more sensitive to the middle and lower face, whereas network B.3 can finely reflect the development of the nasal bone, jaw bone, and other parts, and network B.2 is more sensitive to the frontal bone and hindbrain. Many cases will have a variety of facial defects that are frequently omitted due to doctors’ inattention and lack of knowledge. People with Down syndrome, for example, will have nasal bone loss, ossification of the forehead and maxilla, and facial abnormalities such as ear defects. Such abnormalities can be found in other genetic diseases as well. As a result, our model can not only improve detection rates, but also generate an innovative, interpretable heatmap. This interpretability feature could make it more useful in complementary medicine.

In the research domain, potential applications include: (1) studying the facial abnormalities of fetal genetic diseases and analyzing the facial risk areas; (2) discovering new genetic diseases; and (3) performing a correlation analysis between genetic diseases and facial characteristics. In the clinical domain, potential applications in the future could include: (1) It can be packaged into low-cost screening software and deployed to the cloud or server, which would be a convenient and cost-effective approach for promoting the early detection of genetic disease; (2) deployed to hospitals at all levels for graded diagnosis and treatment to reduce medical errors and medical waste; (3) it provides suggestions for diagnoses for doctors to consider as a complementary diagnostic technique; and (4) large-scale, cross-regional genetic disease screening.

Although the present study proves the potential of our Fgds-EL in screening genetic diseases, the model has several limitations, which we wish to address in the near future: (1) Our study only used a small number of cases of genetic diseases from one center, which may affect the robustness of the model. In the next study, we will collect data from different races (e.g., Black, Latinx, Caucasian, etc.) to improve the robustness of the model. (2) In addition to the cases included in this study, there are many rare genetic diseases with typical facial abnormalities. The next study needs to combine multiple institutions to collect these rare cases. (3) Only one standard section of ultrasonic images is used in this study. In the following study, we will explore the use of other standard sections for genetic disease screening. In addition, while our model appears well suited for a screening purpose (discerning facial abnormalities in genetic diseases), it cannot provide a specific diagnosis based on images. We expect to collect more ultrasonic facial images of genetic diseases in each category and then develop an AI model to realize this function. (4) We did not compare the differences between our model and the integrated test and the NIPT in this experiment, we hope to collect data for comparative experiments in future studies. (5) The model has been tested against three human raters, but the number of human raters involved is relatively small. In the future, we will conduct large-scale comparative research.

In conclusion, we created Fgds-EL, a deep learning model based on the ensemble learning framework, to screen for fetal genetic diseases. Fgds-EL could also predict a risk score for trisomy 21, trisomy 18, trisomy 13, and other rare genetic diseases in an automated, accurate, and quantitative manner. As a new technology for clinic grading, this AI model can assist medical institutions in conducting large-scale fetal genetic disease screening while also reducing the use of medical resources.

## Figures and Tables

**Table 1:** Characteristics of the development set and the test set.

	Development (Training) Set	Test Set
No. of pregnancies with genetic anomaly	111	
No. of normal pregnancies	556	
Ultrasonic equipment	GE Volution E10	
Institution	Guangzhou Women and Children’s Medical Center	
Total no. of qualified ultrasound images	776	156
Genetic disease images	189	63
Normal pregnancies images	587	93

**Table 2:** Fgds-EL performance in screening and identifying genetic diseases.

	AUROC (95% CI)	Sensitivity (95% CI)	Specificity (95% CI)	F1
Fgds-EL	0.986 (0.984–0.987)	0.92 (0.82–0.97)	0.97 (0.90–0.99)	0.935
Network A	0.857 (0.849–0.861)	0.76 (0.64–0.86)	0.81 (0.71–0.88)	0.744
Network B.1	0.889 (0.884–0.896)	0.78 (0.65–0.87)	0.86 (0.77–0.92)	0.784
Network B.2	0.660 (0.654–0.671)	0.70 (0.57–0.80)	0.63 (0.52–0.72)	0.620
Network B.3	0.711 (0.703–0.721)	0.52 (0.40–0.65)	0.93 (0.85–0.97)	0.641

Data are metric value or metric value (95% CI). AUROC = area under the receiver operating characteristics curve.

**Table 3:** Performance of four deep learning algorithms in the test datasets.

	AUROC (95% CI)	Sensitivity (95% CI)	Specificity (95% CI)	F1	Loading Time (Seconds)	Prediction Time (Seconds)
Fgds-EL	0.986 (0.984–0.987)	0.92 (0.82–0.97)	0.97 (0.90–0.99)	0.935	2.638	0.942
ResNet-50	0.870 (0.862–0.875)	0.76 (0.64–0.86)	0.85 (0.76–0.91)	0.768	1.535	0.503
DenseNet-169	0.920 (0.915–0.926)	0.89 (0.78–0.95)	0.88 (0.80–0.94)	0.862	3.167	1.446
DenseNet-201	0.909 (0.907–0.915)	0.73 (0.60–0.83)	0.91 (0.83–0.96)	0.786	3.843	1.744
VGG-16	0.818 (0.812–0.828)	0.70 (0.57–0.80)	0.95 (0.87–0.98)	0.786	1.377	0.102
ResNest50	0.959 (0.956–0.963)	0.889 (0.778–0.950)	0.883 (0.796–0.937)	0.862	24.457	2.232
EfficientFormer	0.890 (0.883–0.894)	0.714 (0.585–0.818)	0.926 (0.848–0.967)	0.783	5.050	0.956
Swin Transformer	0.915 (0.912–0.921)	0.857 (0.741–0.929)	0.830 (0.735–0.897)	0.812	0.931	0.211
RepLKNet	0.916 (0.912–0.922)	0.857 (0.741–0.929)	0.840 (0.747–0.905)	0.818	34.128	1.534

Data are metric value or metric value (95% CI). AUROC = area under the receiver operating characteristics curve.

**Table 4:** The screening performance of the Fgds-EL model and three sonographers.

	Fgds-EL	Junior	Attending	Senior
Accuracy	0.93	0.63	0.74	0.91
Sensitivity (95% CI)	0.91 (0.78–0.97)	0.58 (0.42–0.72)	0.84 (0.70–0.93)	0.91 (0.78–0.97)
Specificity (95% CI)	0.95 (0.84–0.98)	0.67 (0.53–0.79)	0.65 (0.51–0.77)	0.91 (0.79–0.97)

## Data Availability

The data generated and/or analyzed during the current study are available upon reasonable request from the corresponding author. The data can be accessed only for research purposes. Researchers interested in using our data must provide a summary of the research they intend to conduct. The reviews will be completed within 2 weeks and then a decision will be sent to the applicant. The data are not publicly available due to hospital regulation restrictions.

## Supplementary Materials

The following supporting information can be downloaded at: https://www.mdpi.com/article/10.3390/ijerph20032377/s1, Supplemental Material S1, Figure S1: Structure of Network B.2 and Network B.3. ResBlock 1 and ResBlock 2 are two residual modules, and ×n represents n repetitions. A BatchNormalization layer follows each convolutional layer, and all the activation function are uesd ReLU. Supplemental Material S2: Other clinical findings.
