# Supplementary material for: The Two-Stage Ensemble Learning Model Based on Aggregated Facial Features in Screening for Fetal Genetic Diseases
Source: Int J Environ Res Public Health. 2023 Jan 29;20(3):2377. doi: 10.3390/ijerph20032377 (PMC9914999; doi:10.3390/ijerph20032377)
Supplement: Supplementary file 1 [file ijerph-20-02377-s001.zip › ijerph-2134348-supplementary.pdf]

Supplemental Material S1

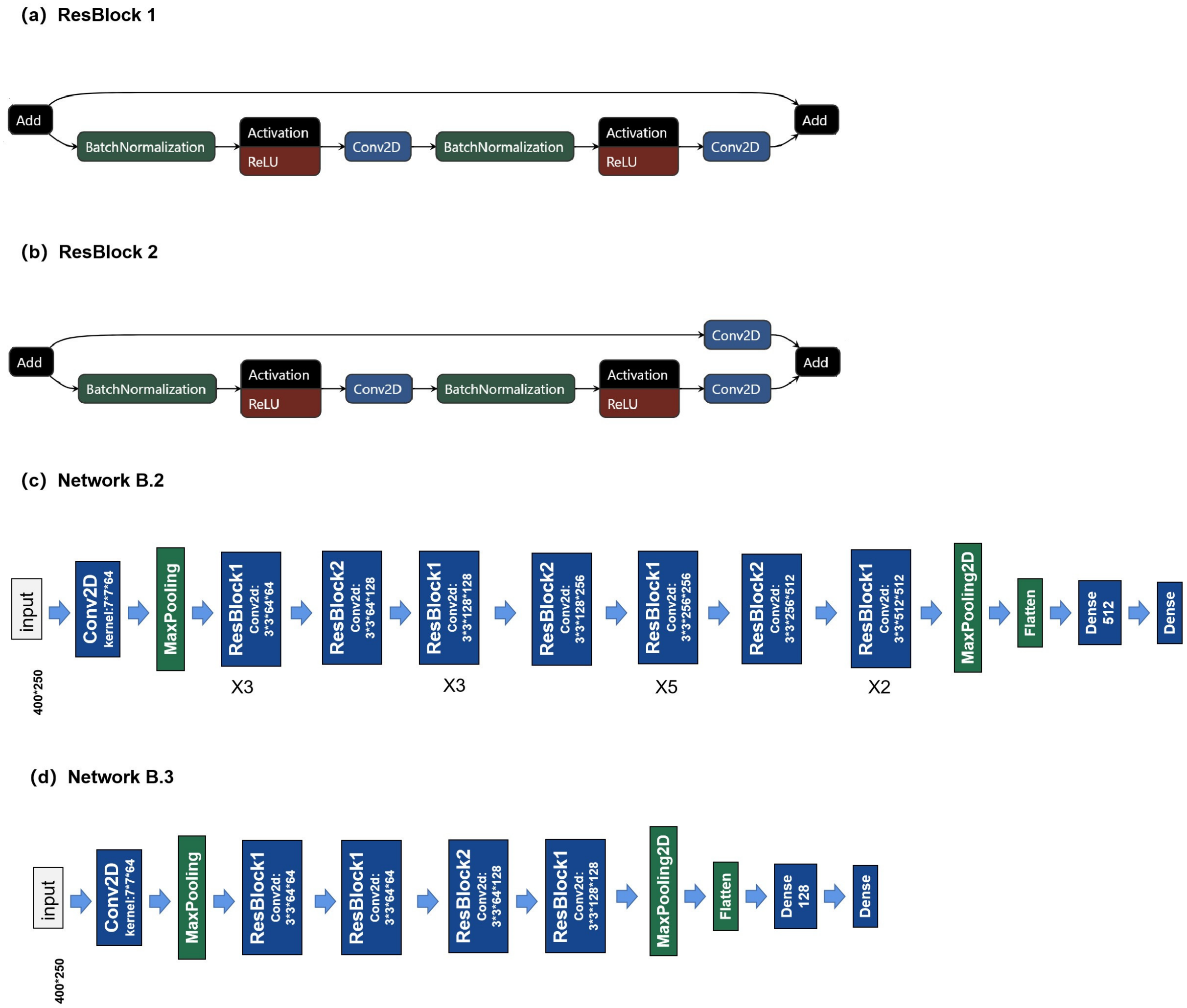

Figure S1. Structure of Network B.2 and Network B.3. ResBlock 1 and ResBlock 2 are two residual modules, and × n represents n repetitions. A BatchNormalization layer follows each convolutional layer, and all the activation function are uesd Relu.

Supplemental Material S2

Other clinical findings

(1) The data indicated one 1q21.1 microdeletion case of ultrasound screening in the second trimester, while the NT screening and the Down screening were normal. Fgds-EL detected the abnormal signal in the middle face area, notably in the connecting area between the nose and eyes. Relevant literature also confirmed that adults of such patients exhibit mild dysmorphic facial features (frontal bossing, deep-set eyes, bulbous nose). (2) In one 15q11-q13 duplication syndrome case, the ultrasound screening in the second trimester, the NT screening and the Down syndrome screening were normal, but Fgds-EL detected the abnormal signal in the nose and mouth area. Relevant studies have also reported that the genetic disease has the facial characteristics of the broad nasal bridge and epicanthal folds in adults. (3) The heatmap of the case with the 15q26.1-q26.3 deletion and the 20p13 duplication revealed abnormal signals in the nasal bone region and the frontal region. (4) The heatmap of the case with the 17q22 microdeletion exhibited abnormal signals in the forehead and mouth areas. (5) The Pyruvate dehydrogenase E1-alpha deficiency case exhibited a difference in the nose and mouth areas. (6) In the screening test of a Helsmoortel-van der AA syndrome, Fgds-EL accurately detected the prominent forehead, wide and depressed nasal bridge, and upturned nasal tip.
